# *LEIGC* long non-coding RNA acts as a tumor suppressor in gastric carcinoma by inhibiting the epithelial-to-mesenchymal transition

**DOI:** 10.1186/1471-2407-14-932

**Published:** 2014-12-11

**Authors:** Yuehua Han, Jun Ye, Dang Wu, Pin Wu, Zhigang Chen, Jian Chen, Shunliang Gao, Jian Huang

**Affiliations:** Department of Gastroenterology, Second Affiliated Hospital, Zhejiang University School of Medicine, Zhejiang University, Hangzhou, 310009 China; Cancer Institute, Second Affiliated Hospital, Zhejiang University School of Medicine, Zhejiang University, Hangzhou, 310009 China; Department of General Surgery, Second Affiliated Hospital, Zhejiang University School of Medicine, Zhejiang University, Hangzhou, 310009 China; Department of Oncology, Second Affiliated Hospital, Zhejiang University School of Medicine, Zhejiang University, Hangzhou, 310009 China

**Keywords:** Long non-coding RNA, Tumor suppressor, Gastric carcinoma, Epithelial-to-mesenchymal transition

## Abstract

**Background:**

Long non-coding RNAs have been shown to have critical regulatory roles in cancer biology. However, the contributions of lncRNAs to gastric cancer remain largely unknown.

**Methods:**

The differential expression of lncRNAs in gastric cancer and paired non-cancerous tissues were identified by microarray and validated using quantitative real-time PCR. Gastric samples from patients with gastric cancer were further analyzed for levels of a specifically downregulated lncRNA (termed as *LEIGC*).

**Results:**

We found that there were significantly lower levels of *LEIGC* expression in cancer tissue than in adjacent non-cancerous tissues in human gastric cancers (*P* < 0.01). Overexpression of *LEIGC* suppressed tumor growth and cell proliferation, and enhanced the sensitivity of gastric cancer cells to 5-fluorouracil (5-FU), whereas knockdown of *LEIGC* showed the opposite effect. We further demonstrated *LEIGC* functions by inhibiting the epithelial-to-mesenchymal transition (EMT) in gastric cancer.

**Conclusions:**

Our data suggested that *LEIGC* is a tumor-suppressing lncRNA in gastric cancer, and led us to propose that lncRNAs may play important regulatory roles in cancer development and progression.

**Electronic supplementary material:**

The online version of this article (doi:10.1186/1471-2407-14-932) contains supplementary material, which is available to authorized users.

## Background

Gastric cancer is the fourth leading cause of cancer death, with a high mortality worldwide, especially in Asia [1, 2]. Unfortunately, gastric cancer is difficult to cure unless it is identified at an early stage, before it has begun to spread. The 5-year survival rate of gastric cancer patients remains poor, at approximately 40%, despite recent advances in surgical techniques and medical treatment [3, 4]. Metastasis is the main cause of death from such tumors. Thus, there is an urgent need to identify new molecular markers for early diagnosis, prediction of metastatic progression and prognosis of gastric cancer patients.

The human transcriptome comprises not only large numbers of protein-coding messenger RNAs (mRNAs), but also many non-protein coding transcripts that function as important regulatory molecules in tumor suppressor or oncogenic pathways [5]. Non-coding RNAs are divided into short non-coding RNAs and long non-coding RNAs depending on their length. Long non-coding RNAs (lncRNAs) are defined as non-coding RNAs of more than 200 nucleotides in length, and are characterized by the complexity and diversity of their sequences and mechanisms of action [6]. Recent deep transcriptome sequencing and microarray studies have revealed that 70–90% of the human genome is estimated to be transcribed into mostly non-protein-coding RNA [7]. Increasing evidence indicates that lncRNAs exert important roles in a wide range of biological processes, including cell differentiation, chromatin remodeling, immune responses and tumorigenesis [6–8]. LncRNA levels are strongly associated with aberrant gene expression that may drive cancer development and progression [9], such as *HOTAIR* in non-small cell lung cancer (NSCLC) [10], *PRNCR1* (also known as *PCAT8*) and *PCGEM1* in prostate cancer [11], and *MEG3* in cervical cancer and meningiomas [12, 13]. Thus, differential expression of lncRNAs may be profiled to aid cancer diagnosis, prognosis and selection of potential therapeutics.

Although lncRNAs play important roles in human diseases, the mechanism through which they contribute to cancer development is still largely unknown. LncRNAs can regulate critical cancer pathways at a transcriptional, post-transcriptional and epigenetic level [14]. Mounting evidence suggests that a major role of lncRNAs is to act as modular scaffolds for protein-chromatin interactions [15]. Several lncRNAs can control gene expression by direct recruitment of histone-modifying enzymes to chromatin [6, 15]. Chromatin modification and DNA methylation are crucial epigenetic events that are fundamentally disturbed during the development of cancer. LncRNAs can also affect protein-coding transcript response to different biological processes [16].

However, there are only preliminary studies on the role of lncRNAs in gastric cancer [17–19], and the overall pathophysiological contributions of lncRNAs to gastric cancer remain largely unknown. A current estimate of the lncRNA gene number in the human genome ranges from 8000–20,000 unique lncRNAs [20, 21], suggesting lncRNAs constitute a large yet undiscovered part of normal cellular networks that may be disrupted in cancer. Therefore, it is of great importance to explore the molecular mechanisms of lncRNAs in gastric cancer development and progression. In this study, we aimed to investigate the expression pattern and clinicopathological implications of lncRNAs in gastric cancer tissues. We identified a new specific differentially-expressed lncRNA (termed *LEIGC*), which was downregulated in gastric cancer tissues compared with adjacent non-cancerous tissues. Then we performed gain- and loss-of-function studies to determine the effect of *LEIGC* on tumor growth, cell proliferation, and migration, and showed that *LEIGC* suppressed tumor growth, cell proliferation and EMT in gastric cancer, and increased the sensitivity of gastric cancer cells to 5-FU.

## Methods

### Cell lines

Human gastric cancer cell lines, MGC-803, AGS, SGC-7901 were purchased from the cell bank of China Academy of Medical Science (China). Cells were cultured in RPMI 1640 medium (Gibco, Carlsbad, CA, USA) supplemented with 10% fetal bovine serum (FBS, Gibco) and maintained at 37°C with 5% CO_2_.

### LncRNA expression microarray analysis

Total RNA of gastric cancer tissues and paired normal tissues were extracted using Trizol reagent (Invitrogen, Carlsbad, CA, USA) and treated with RNase-free DNase I (Qiagen, Valencia, CA, USA) according to the manufacturer’s protocol. The quantity and quality of RNA was evaluated using a Nanodrop spectrophotometer (Thermo Scientific, Worcester, MA, USA). The lncRNA expression profile of each sample was examined using a lncRNA expression microarray (SurePrint Human Gene Expression Microarray Kit, Agilent technologies, Santa Clara, CA, USA). The BROAD Institute database was used in the genesis of the array. After hybridization and washing, the processed slides were scanned with the Agilent Microarray Scanner (Agilent technologies Santa Clara, CA, USA). Raw data were extracted as pair files using Feature Extraction software 10.7 (Agilent technologies). A fold change of ≥ 2.0 or <0.5 (*P* ≤ 0.05) was set as a threshold for up- and downregulated genes, respectively, and data were presented as mean ± SD. Raw data were normalized by a Quantile algorithm using Gene Spring Software 11.0 (Agilent technologies). Hierarchical clustering was performed based on differentially-expressed lncRNAs using Cluster Treeview software from Stanford University.

### Structural analysis of *LEIGC*

We used the BLAT program of the University of California Santa Cruz (UCSC), Genome Browser, BLAST, and MAP VIEW of the NCBI to analyze the gene sequence and chromosomal location of *LEIGC*.

### Quantitative real time PCR analysis

Total RNA from cell lines and tissues was purified by the Trizol (Invitrogen) method according to the manufacturer’s instructions. RNA quantity and quality were evaluated using a Nanodrop spectrophotometer. The RNA was reverse-transcribed into cDNA using the Reverse Transcriptase M-MLV (Promega, Madison, WI, USA), and the expression of *LEIGC*, *snail*, *zeb*, *slug*, *CDH1*, and *twist* was measured using SYBR Green PCR Master Mix (Applied Biosystems, Foster City, CA, USA) on the Stepone plus system (Applied Biosystems). Each sample was run in triplicate and the gene expression levels were normalized to *GAPDH* expression. The primers for quantitative real time PCR (qRT-PCR) analysis were listed in Table 1.

**Table 1:** **Primer sequences used in qRT-PCR**

Gene name	Primers
LEIGC	F: 5’- agg ata cgt aag aaa cac ttc tgt -3’
	R: 5’- tgt ctt ggt tta aca acc ga -3’
*snail*	F: 5’- acc cca cat cct tct cac tg −3
	R: 5’- tac aaa aac cca cgc aga ca −3
*slug*	F:5’- aca cac aca cac cca cag ag −3
	R:5’- aaa tga ttt ggc agc aat gt −3
*zeb*	F: 5’- gca caa cca agt gca gaa ga -3’
	R: 5’- cat ttg cag att gag gct ga -3’
*E-cadherin*	F: 5’- tgc tct tgc tgt ttc ttc gg-3’
	R: 5’- tgc ccc att cgt tca agt ag-3’
*N-cadherin*	F: 5’- tgg atg gac ctt atg ttg ct -3’
	R: 5’- aac acc tgt ctt ggg atc aa -3’
*twist*	F: 5’-gga gtc cgc agt ctt acg ag-3’
	R: 5’-cca gct tga ggg tct gaa tc-3’
GAPDH	F: 5’-ctt agc acc cct ggc caa g-3’
	R:5’-gat gtt ctg gag agc ccc g-3’

Cycling conditions were 10 min at 95°C for initial denaturation, followed by 40 cycles of 15 sec at 95°C for denaturation, 30 sec at 60°C for combined annealing and 30 sec at 72°C for primer extension. Each sample was run in triplicate and the gene expression levels were normalized to that of *GAPDH* expression.

### Establishment of lncRNA or lncRNA-shRNA stable cells

*LEIGC* vector (LV6-Puro) or *LEIGC*-shRNA vector (pGLV2-U6-Puro) (Additional file 1: Figure S1) and scrambled shRNA or non-related lncRNA vector lentiviral particles (GenePharma Tech, Shanghai, China) were transfected into MGC-803 cells. Cells were selected with 5 μg/ml puromycin (Sangon Biotech, Shanghai, China) at 48 h after transfection. The overexpression and knockdown efficiencies were verified by qRT-PCR.

### *In vitro*motility assay

Transwell insert chambers with 8-μm porous membranes (Corning Incorporated, NY, USA) were used for motility assays. Cells were washed three times with PBS and 5 × 10^4^ cells in serum-free media were added to the top chamber. The bottom chamber was filled with RPMI 1640 medium containing 15% FBS. Cells were incubated for 24 h at 37°C in 5% CO_2_. To quantify migrating cells, cells in the top chamber were removed using a cotton-tipped swab, and the migrated cells were fixed in methanol and stained with 0.1% crystal violet.

### Cell growth assay

Cells were seeded at a density of 3 × 10^3^ cells/well in a 96-well plate containing 0.2 ml RPMI 1640 medium with 10% FBS. Then 20 μl MTS (3-[4,5-dimethylthiazol-2-yl] -5-[3-carboxmethoxy phenyl]-2-[4-sulfophenyl]-2H-tetrazolium salt) (Promega) reagent was added to each well and the cells were incubated at 37°C for 4 h. The OD values were measured at 490 nm on a microplate reader (Bio-Rad, Hercules, CA, USA) and assessed daily for 7 days.

### Colony formation assay

The proliferative ability of cells was tested in colony formation assays. Approximately 300 cells were seeded into each well of a 12-well plate. After incubation at 37°C for 14 days, the cells were washed twice with PBS, fixed with methanol and then stained with 0.1% crystal violet. The number of colonies containing more than 300 cells was counted under a microscope.

### Tumorigenesis assay

A total of 1 × 10^6^ cells suspended in 100 μl RPMI 1640 were implanted into the hindquarters of 4-week-old female NOD/SCID mice to assess their ability to initiate tumor xenografts. The use and care of experimental animal was approved by Institutional Animal Care and Use Committee of Zhejiang Chinese Medical University. Tumors were measured weekly and their volume calculated as length × width × width/2 [22].

### Western blotting

Total protein from cells was lysed using M-PER Mammalian Protein Extraction Reagent (Thermo) supplemented with a protease inhibitor cocktail (Sigma, St Louis, MO, USA). Samples were denatured, and equal amounts of protein were subjected to SDS-PAGE, and then transferred to nitrocellulose membrane. After blocking with 5% non-fat milk in TBST for 60 min, membranes were incubated with primary antibody dissolved in 5% bovine serum albumin in TBST overnight at 4°C. The following primary antibodies were used: anti-human- E-cadherin (1:2000, 24E10; Cell Signaling Technology, Danvers, MA, USA), anti-human-Vimentin (1:2000, D21H3; Cell Signaling Technology), anti-human-Snail (1:1000, C15D3; Cell Signaling Technology), anti-human-Twist (1:1000; Cell Signaling Technology), anti-human-Zeb (1:1000, D80D3; Cell Signaling Technology), and anti-human-Slug (1:1000, C19G7; Cell Signaling). Human GAPDH (1:5000; KangChen, Shanghai, China) was used as an internal reference.

### Cell viability assay

MGC-803 cells were transfected with *LEIGC* vector or *LEIGC* shRNA vector and scrambled shRNA or non-related lncRNA vector, and incubated for 24 h. Then cells were reseeded in 96-well plates and treated with 5-FU (NanTong Pharmaceutical Factory, China) at different concentrations for 48 h. The cell viability was determined by the MTT assay: 20 μl of MTT (Sigma) per well was added and incubated at 37°C for 4 h. The reaction was stopped by adding 200 μl of dimethyl sulfoxide (DMSO) to each well followed by measuring the absorbance at 570 nm on a microplate reader (Bio-Rad, USA) for the indicated time periods at 37°C to determine the individual IC50 values (50% cell growth inhibitory concentrations).

### Clinical gastric cancer sample analysis

The study was approved by the Research Ethics Committee of Zhejiang Medical University, China. Human gastric cancer and paired normal tissues were obtained in accordance with the ethical standards of the institutional committee. All gastric cancer patients gave written informed consent for the use of clinical specimens in medical research. Specimens were collected between 2007 and 2009 at the Second Affiliated Hospital, Zhejiang University School of Medicine. The diagnosis of each case was confirmed independently by two pathologists. Tumors were staged using the Union Internationale Contre le Cancer (UICC) staging system (Table 2).

**Table 2:** **Clinicopathological features of gastric cancer patients**

Parameters	Cases (n = 35)
Gender	
Male	26
Female	9
Age (years)	
Male	63 (42–87)
Female	58 (31–71)
Histological grade	
Well differentiated	4
Moderate differentiated	24
Poor differentiated	7
Lymph node metastasis	
N0	10
N1,2,3	25
Distant metastasis	
M0 (NM group)	18
M1 (M group)	17
TNM stage	
I	7
II	4
III	19
IV	5

*TNM stage* tumor-node-metastasis stage.

### Statistical analysis

Data are presented as the means ± standard error of the mean (SEM). All statistical analyses were performed using SPSS 16.0 software. The qRT-PCR results from paired clinical samples were analyzed by a two-tail paired Student’s t-test and the other results by a two-tail unpaired Student’s t-test. *P* values of <0.05 indicated statistical significance.

## Results

### Expression of *LEIGC*in gastric cancer tissues

**Figure 1:**
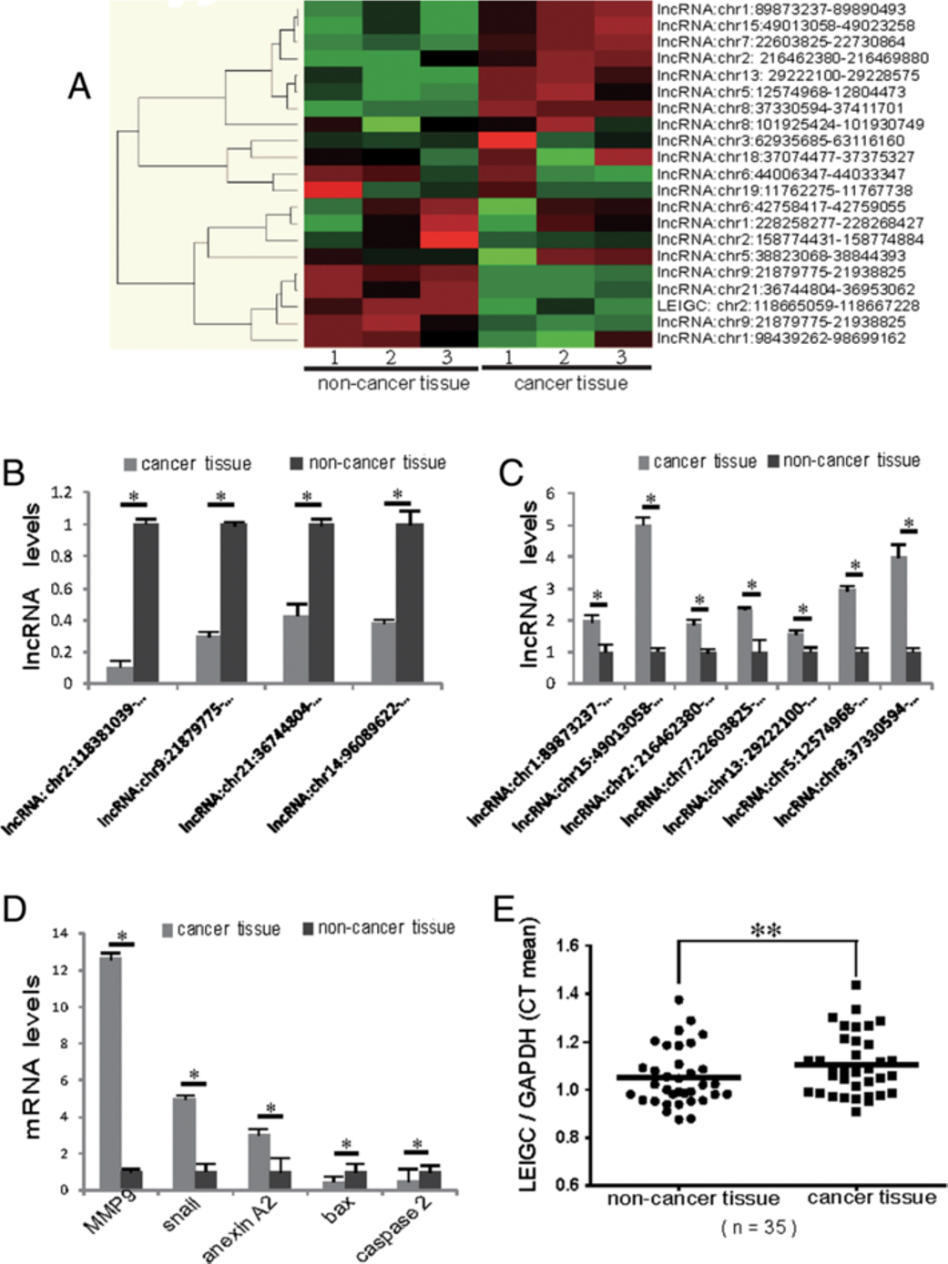
**Alterations in lncRNA expression profiles between gastric tumor tissues and paired adjacent non-tumorous tissues. (A)**. lncRNA expression was evaluated by an lncRNA expression microarray. Results from hierarchical clustering showed different lncRNA expression among samples. “Red” indicates high expression; “green” indicates low expression. **(B)**. Results from qRT-PCR experiments demonstrating downregulated expression of lncRNAs in three gastric cancer samples. **(C)**. qRT-PCR analysis of lncRNAs selected from microarray results in three gastric cancer samples revealed upregulated expression. **(D)**. qRT-PCR verification of mRNA selected from microarray results in three gastric cancer samples. **(E)**. Expression of lncRNA *LEIGC* in fresh gastric cancer tissues from 35 patients was detected by qRT-PCR. *LEIGC* levels were normalized to *GAPDH* and expressed in terms of the threshold cycle (CT) ratio. Error bars represent the means ± SEM. *P < 0.05; **P < 0.01.

To identify genes involved in gastric cancer progression, lncRNA array analysis was performed on total RNA isolated from three gastric cancer samples, and matched peri-cancerous samples. Microarray analysis detected different upregulated and downregulated lncRNAs (Figure 1A). To select lncRNA for further studies, we applied more stringent filtering criteria: (1) high-expression levels; and (2) similar expression patterns in different clinical samples. Results demonstrated there were four significantly downregulated lncRNAs (lncRNA: chr2:118381039–118383698, lncRNA:chr9:21879775–21938825, lncRNA:chr21:36744804–36953062, lncRNA: chr14:96089622–96112397) and seven significantly upregulated lncRNA (lncRNA:chr1:89873237–89890493, lncRNA:chr15:49013058–49023258, lncRNA: chr2:216462380–216469880, lncRNA:chr7:22603825–22730864, lncRNA:chr13: 29222100–29228575, lncRNA:chr5:12574968–12804473, lncRNA:chr8: 37330594– 37411701) in gastric cancer tissues versus paired normal tissues (*P* < 0.05; Figure 1A).The expression levels of selected lncRNAs (*P* < 0.05; Figure 1B, C) and mRNA (*P* < 0.05; Figure 1D) were also validated by qRT-PCR analyses on the same three human gastric cancer tissue samples and paired non-cancerous tissues. We further examined lncRNA: chr2:118381039–118383698 expression levels in 35 paired gastric cancer samples and adjacent normal tissues by qRT-PCR. We observed that lncRNA: chr2:118381039–118383698 levels were significantly downregulated in gastric cancer tissue samples compared with normal tissue samples (*P* < 0.01; Figure 1E); this was named as *LEIGC*. These data indicated that *LEIGC* may be a probable new target to prevent relapse and metastasis of gastric cancer.

### Structural analysis of *LEIGC*

**Figure 2:**
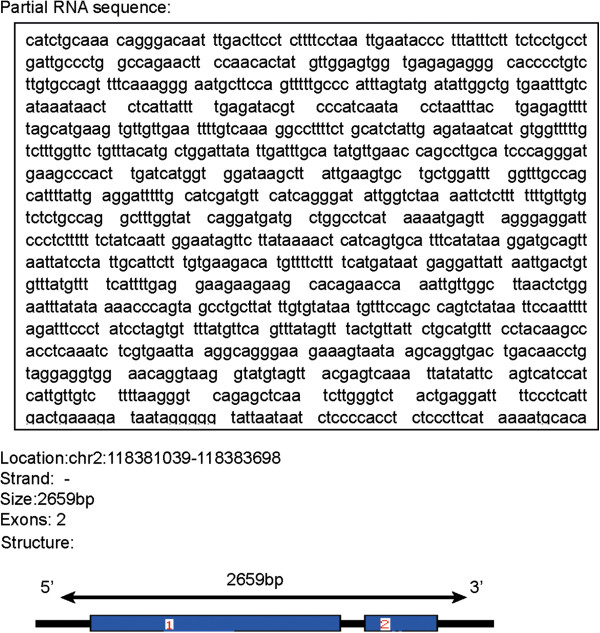
**Partial gene sequence and molecular structure map of** ***LEIGC.*** Gene sequence was analyzed by BLAT, BLAST and MAP VIEW programs. Map of *LEIGC* molecular structure was revealed by the BLAT program; 1 and 2 indicate different exons.

We named this lncRNA gene as *LEIGC* (lower expression in gastric cancer) according to the human gene nomenclature guideline by the Human Gene Nomenclature Committee (HGNC) [23]. We found no repetitive naming compared with other genes in Genbank and EMBO datasets by BLAST. Gene sequence analysis by BLAT, BLAST and MAP VIEW of NCBI revealed that *LEIGC* was present in a novel amplicon on chromosome 2 and located at 2q14.1 (Figure 2). *LEIGC* consists of 2659 bp with two exons (Figure 2).

### *LEIGC*suppresses tumor growth *in vitro*

**Figure 3:**
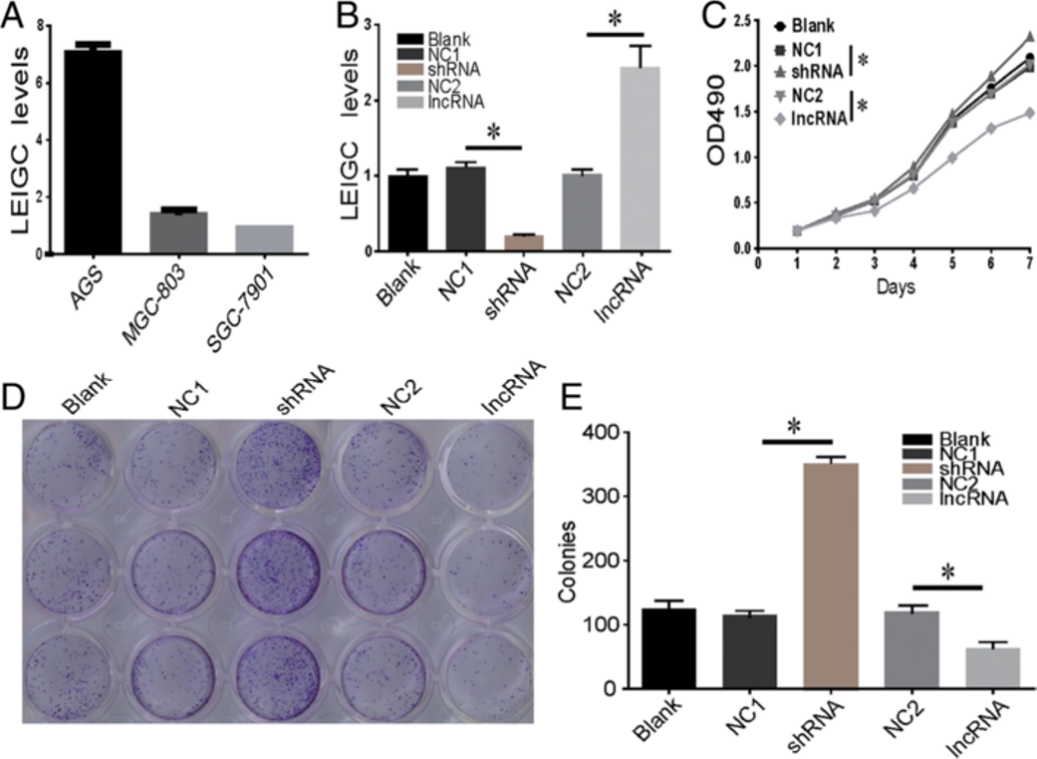
**Growth and colony formation assays of MGC-803 cells following overexpression or knockdown of** ***LEIGC.*** Cells were transfected with *LEIGC*-shRNA vector (shRNA) and scrambled shRNA vector (NC1), or *LEIGC* vector (lncRNA) and non-related lncRNA vector (NC2), and blank control (blank). **(A)**. Basal levels of *LEIGC* in MGC-803, SGC-7901 and AGS cells. **(B)**. Knockdown and overexpression of *LEIGC* was confirmed by qRT-PCR. **(C)**. Cell proliferation rate was determined by measuring the absorbance at 490 nm in MTS assays. **(D and E)**. Colony formation assays of MGC 803 cells. *P>0.05.

To examine the effect of *LEIGC* overexpression and knockdown in gastric cell proliferation, we performed MTS and colony formation assays. MGC-803 cells are a gastric cancer cell line with moderate *LEIGC* expression level, as confirmed by qRT-PCR in our study (Figure 3A). We stably transfected *LEIGC* vector (LV6-Puro), *LEIGC*-shRNA vector (pGLV2-U6-Puro) and their control vector lentiviral particles into MGC-803 cells. The efficiency of overexpression and knockdown was verified by qRT-PCR (Figure 3B). Overexpression of *LEIGC* in MGC-803 cells markedly reduced the number of cell colonies formed (Figure 3E). When compared with cells transfected with non-related lncRNA vector, overexpression of *LEIGC* significantly decreased the cell proliferation rate, as measured by MTS (*P* < 0.05; Figure 3C). In contrast, *LEIGC* knockdown in MGC-803 cells showed the opposite results (*P* < 0.05; Figure 3C). These data supported the tumor suppressive function of *LEIGC* in gastric cancer cells.

### *LEIGC*inhibits migration of gastric cancer cells *in vitro*

**Figure 4:**
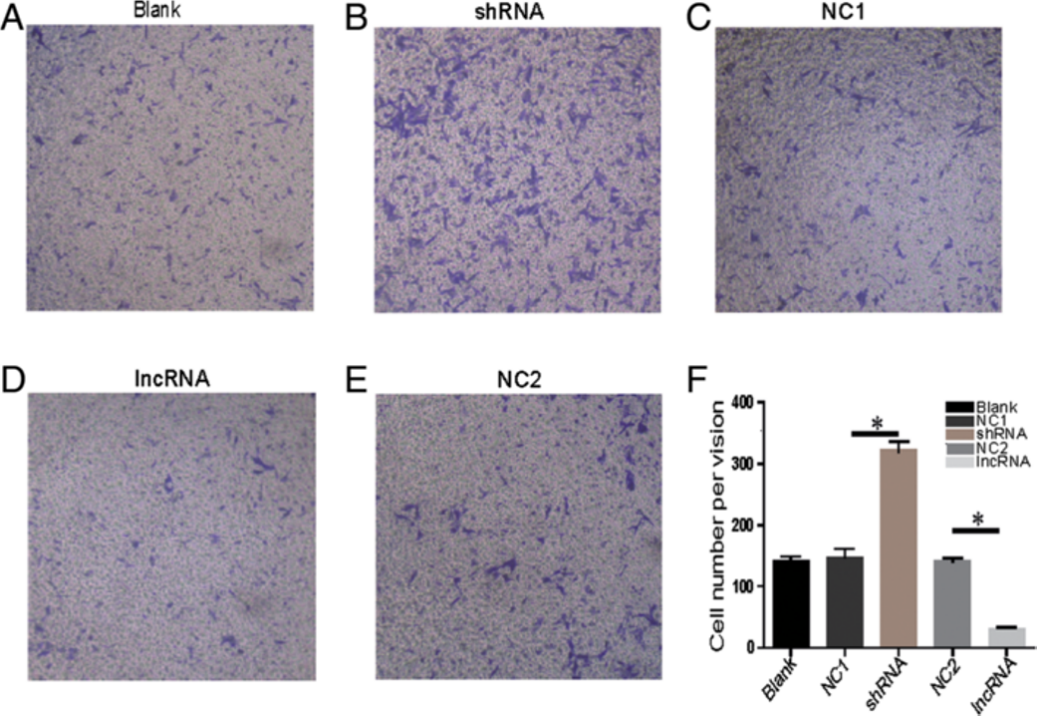
**Effect of LEIGC knockdown and overexpression on cell migration. (A)**. MGC-803 cells that had migrated to the bottom chamber after transfection with blank vector (blank); **(B and C)**. MGC-803 cells that had migrated to the bottom chamber after transfection with shRNA vector (shRNA) and scrambled shRNA vector (NC1); **(D and E)**. MGC-803 cells that had migrated to the bottom chamber after transfection with *LEIGC* vector (lncRNA) and non-related lncRNA vector (NC2); **(F)**. Quantification of different MGC-803 cells that had migrated to the top chamber *P<0.05.

The important process of carcinoma progression is that dissociated epithelial cells acquire migration and invasive abilities and can pass through the basement membrane to distant tissues. To determine whether *LEIGC* regulates the migratory ability of gastric cancer cells, we performed migration assays. We used MGC-803 cells as a model because of their strong motility. Pooled *LEIGC*-overexpressing cells showed a significantly lower migration potential than *LEIGC* knockdown cells and controls in the migration assay (Figure 4).

### *LEIGC*knockdown promotes tumor progression *in vivo*

**Figure 5:**
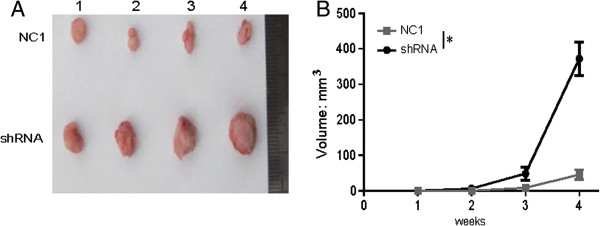
***LEIGC*** **knockdown enhanced the tumorigenic potential of gastric carcinoma cells** ***in vivo.*** **(A)**. Exposure of tumors inoculated with *LEIGC*-shRNA vector cells (shRNA) and scrambled shRNA vector (NC1) when mice were sacrificed. **(A)**. Representative image of xenograft tumors in NOD/SCID mice subcutaneously injected with MGC-803 cells; 1, 2, 3, and 4 indicate the different mice. **(B)**. Comparison of xenograft formation *in vivo*. Tumor volumes were measured each week. Error bars represent means ± SEM, *P < 0.05.

We examined the progression potential of *LEIGC* knockdown in MGC-803 cells using a NOD/SCID mouse model. MGC-803 cells transfected with *LEIGC*-shRNA or scrambled vectors were subcutaneously injected into NOD/SCID mice (n = 4). Tumor growth was monitored by standard caliper measurement in a blinded fashion. Tumors formed in sites injected with MGC-803 cells (Figure 5). After 4 weeks, animals were sacrificed for determination of tumor weights. Histopathological examination demonstrated that MGC-803 cells with or without *LEIGC* knockdown generated uniform implanted tumors. As shown in Figure 5B, tumor development was first visible at 14 days after injection. Tumors of MGC-803 cells transfected with scrambled vector grew significantly slower in comparison with tumors of MGC-803 cells transfected with *LEIGC*-shRNA vector (*P* < 0.05; Figure 5B). In addition, MGC-803 cells transfected with *LEIGC*-shRNA vectors generated tumors that were significantly larger than those derived from control cells at the time of resection (*P* < 0.05; Figure 5A). Thus, *LEIGC* knockdown in MGC-803 cells was tumorigenic and resulted in the formation of aggressive tumors that were well palpable.

### *LEIGC*enhances chemosensitivity to 5-FU in gastric cancer

**Figure 6:**
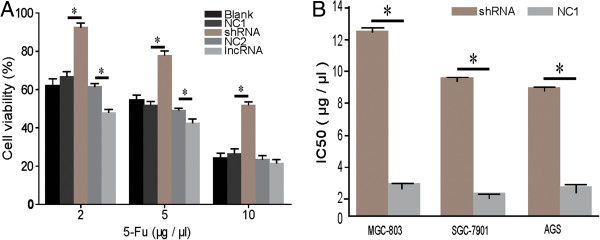
**Effect of** ***LEIGC*** **on gastric cancer cell chemosensitivity to 5-FU. (A)**. MGC-803 cells were seeded in 96-well plates and treated with different concentrations of 5-FU for 48 h. Cell viability was measured using MTT assays. **(B)**. IC50 of gastric cancer cells (SGC 7901, MGC-803 and AGC) transfected with *LEIGC*-shRNA vector (shRNA) and scrambled shRNA vector (NC1), *P < 0.05.

To determine the effect of *LEIGC* on the sensitivity to 5-FU chemotherapeutic agent, cell viability was measured using the MTT assay. Transfection of MGC-803 cells with *LEIGC* vector resulted in significantly decreased cell viability with treatment of 5-FU at 2 and 5 μg/μl (*P* < 0.05; Figure 6A) compared with control cells, whereas there was no obvious change in cell viability at 10 μg/μl. Transfection of MGC-803 cells with *LEIGC*-shRNA vector resulted in significantly increased cell viability in each of the 5-FU treatments (2, 5,10 μg/μl) (*P* < 0.05; Figure 6A). Next we measured the IC50 values for 5-FU following *LEIGC* knockdown in gastric cancer cells (MGC-803, SGC-7901 and AGS) and control cells. The result showed *LEIGC* knockdown cells had the lowest sensitivity to 5-FU (*P* < 0.05; Figure 6B).

### *LEIGC*is a novel factor that prevents EMT in gastric cancer

**Figure 7:**
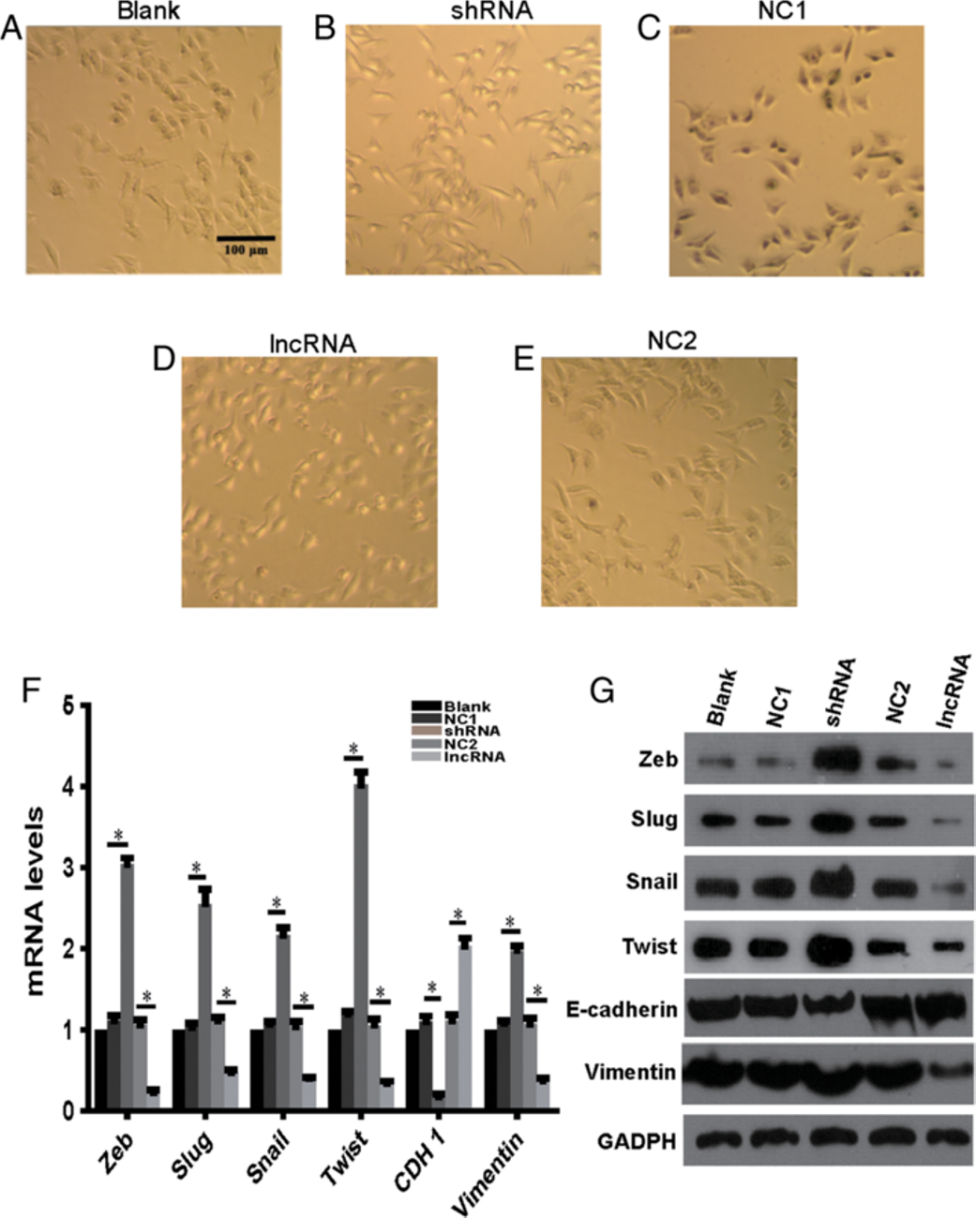
***LEIGC*** **inhibited EMT in MGC-803 cells. (A)**. MGC-803 cells transfected with blank control vector (blank); **(B and C)**. Cells transfected with *LEIGC*-shRNA vector (shRNA) and scrambled shRNA vector (NC1); **(D and E)**. Cells transfected with *LEIGC* vector (lncRNA) and non-related lncRNA vector (NC2). **(F)**. Effects of *LEIGC* on the expression of EMT-related genes *CDH1*, *twist*, *snail*, *slug*, and *zeb* at the mRNA level were analyzed by qRT- PCR. *P<0.05. **(G)**. Effects of *LEIGC* on the expression of EMT-related proteins. After transfection of the cells with different vectors, E-cadherin, Vimentin, Twist, Slug, and Zeb expression levels were determined by western blotting. GAPDH protein levels served as an internal control.

To determine whether *LEIGC* contributes to tumor metastasis, we performed morphological observations of MGC-803 cells following *LEIGC* overexpression and knockdown. Intriguingly, *LEIGC* knockdown cells appeared spindle-shaped and fibroblastic in monolayer cultures, and displayed a clear transition from cobblestone-like cells to spindle-like fibroblastic morphology, whereas *LEIGC*-overexpressing cells maintained their cobblestone-like phenotype (Figure 7). This morphological change implied that the *LEIGC* knockdown cells had undergone trans-differentiation from epithelial cells to mesenchymal cells.

To confirm that *LEIGC* knockdown in MGC-803 cells resulted in a mesenchymal phenotype, we analyzed the gene expression profiles of *LEIGC*-overexpressing cells versus knockdown MGC-803 cells. As shown in Figure 7, the epithelial cell-related gene *CDH1* was significantly downregulated, whereas several mesenchymal cell markers (such as *snail*, *slug*, *zeb*, and *twist*) were significantly upregulated in *LEIGC* knockdown cells compared with *LEIGC*-overexpressing cells (Figure 7F). We further examined EMT-associated protein expression in MGC-803 cells by western blotting. As shown in Figure 7G, *LEIGC* knockdown cells demonstrated lower expression of E-cadherin and higher expression of Vimentin, Snail, Slug, Zeb, and Twist. Overexpression of *LEIGC* showed the opposite effect. These data indicated that *LEIGC* was a potent EMT inhibitor in gastric cancer cells.

## Discussion

Over the past few years, hundreds of lncRNAs have been shown to play important roles in both transcription and post-transcriptional processes. Studies have reported that lncRNA dysfunctions are associated with a broad range of human tumors, including those of metastasis-associated lung adenocarcinoma transcript 1 (*MALAT1*), HOX antisense intergenic RNA (*HOTAIR*), antisense non-coding RNA in the INK4 locus (*ANRIL*), and lncRNA-p21 [10, 24]. lncRNAs are aberrantly expressed in many types of cancers [25, 26]. However, the potential roles of lncRNAs in human cancers are not well understood. In this study, we verified that *LEIGC* was significantly downregulated in gastric cancer tissues compared with paired non-cancerous tissues. Furthermore, our results indicated that *LEIGC* inhibited tumor growth, proliferation, migration, and EMT in gastric cancer cells. Hence, our results also suggest that *LEIGC* is a putative tumor/metastasis suppressor in gastric cancer.

Recently, many studies have shown that lncRNAs have important roles in the regulation of numerous biological processes in cancer, including tumor proliferation, migration, angiogenesis, and EMT. Altered expression of lncRNAs has been documented in different human cancer types, prompting increased interest in their use as biomarkers for diagnosis and prognosis as well as potential therapeutic targets [7]. For example, a study demonstrated that *HULC* was significantly overexpressed in gastric cancer cell lines and gastric cancer tissues compared with normal tissues, and its overexpression was correlated with distant metastasis and lymph node metastasis [17]. Knockdown of *HULC* inhibited proliferation, invasion and EMT, and promoted cell apoptosis in SGC-7901 gastric cancer cells. Recently, increased levels of *HOTAIR* in primary breast tumors were shown to correlate with breast cancer invasiveness and metastasis [25]. *HOTAIR* bridges together the PRC2 complex with the LSD1 H3K4 demethylase complex, and recruits both complexes to target genes to coordinately alter several histone modifications and enforce gene silencing. The increased expression of *HOTAIR* in human gastric cancers was associated with venous invasion, lymph node metastases and a lower overall survival rate [9, 15].

To explore the exact mechanisms of *LEIGC* in gastric cancer, we used gene transfection experiments to overexpress and silence *LEIGC* in MGC-803 gastric cancer cells. The key event for malignant tumor progression is metastasis, which is based on tumor cell migration and invasion. Metastasis accounts for the majority of gastric cancer-related mortality, but the mechanism of the metastatic process in gastric cancer is very complex, and still not completely understood. EMT was originally recognized as a critical step to metazoan embryogenesis and in defining structures during organ development [27]. During the last decade, a number of studies have associated EMT with cancer progression and metastasis in gastric cancer. In our transwell assay, knockdown of *LEIGC* dramatically promoted cell migration in gastric cancer cells (Figure 4). We found *LEIGC* silencing was associated with features typical of EMT, including the conversion of the cobblestone-like epithelial morphology to spindle-shape mesenchymal morphology, reduced expression of *CDH1*, and increased expression of *snail*, *slug*, *twist* and *zeb*. Consistent with the observed morphological changes, some hallmark proteins of epithelial cells were lost or reduced during the transition, such as E-cadherin. In contrast, the mesenchymal protein vimentin was upregulated. It is well known that E-cadherin plays a critical role in the suppression of tumor invasion. Most epithelial cancers display downregulated or inactivated E-cadherin [28, 29]. It has been shown that the restoration of functional E-cadherin suppresses invasion in many tumor types. Snail, Twist, Slug and Zeb associated with EMT have all been shown to target E-boxes on the E-cadherin promoter, repressing its expression [30, 31]. We observed that *snail*, *slug*, *twist* and *zeb* genes and corresponding proteins were highly elevated in *LEIGC* knockdown cells (Figure 7). However, overexpression of *LEIGC* resulted in the opposite effect in MGC-803 cells. Taken together, these data indicated that *LEIGC* is a critical regulator in preventing EMT in gastric cancer. However, in our study, no significant correlation was found between *LEIGC* expression and distant tumor metastasis or lymph node metastasis in gastric cancer (data not shown). This might be because of the low number of gastric patients in our study.

## Conclusions

In summary, our data provides evidence that may mechanistically link the expression of LEIGC to the proliferation and migration of gastric cancer cells. We demonstrate that *LEIGC* functions as a tumor suppressor lncRNA in gastric cancer by inhibiting EMT, and propose that lncRNAs may play important regulatory roles in cancer development and progression. Further analysis and investigation of the mechanisms of *LEIGC* in the molecular etiology of gastric cancer will provide lncRNA-directed diagnostic and therapeutic tools against this deadly disease.

## Electronic supplementary material

Additional file 1: Figure S1: The original information about the vectors 'LV-puro' and ‘pGLV2-U6-puro’. **(A)** The structure of the vector ‘LV-puro’. **(B)** The structure of the vector ‘pGLV2-U6-puro’. (TIFF 646 KB)
