# Supplementary figures and images for: LEIGC long non-coding RNA acts as a tumor suppressor in gastric carcinoma by inhibiting the epithelial-to-mesenchymal transition
Source: BMC Cancer. 2014 Dec 11;14:932. doi: 10.1186/1471-2407-14-932 (PMC4295322; doi:10.1186/1471-2407-14-932)

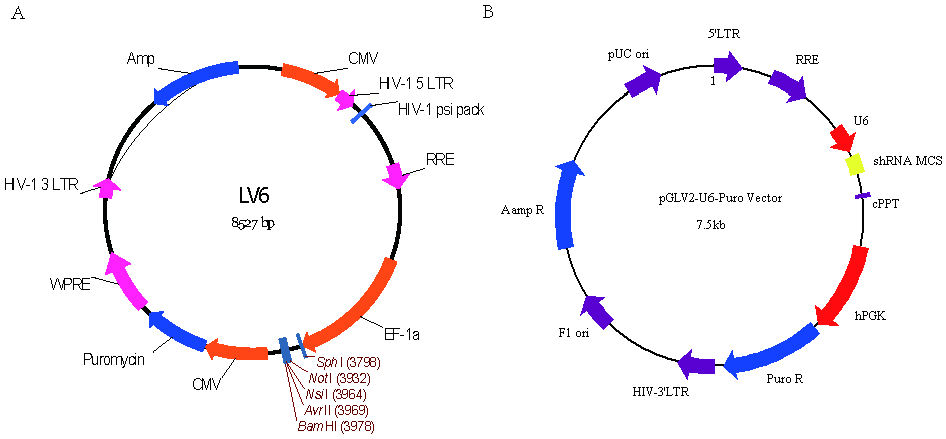

Supplement: Supplementary file 1 — Additional file 1: Figure S1: The original information about the vectors 'LV-puro' and ‘pGLV2-U6-puro’. (A) The structure of the vector ‘LV-puro’. (B) The structure of the vector ‘pGLV2-U6-puro’. (TIFF 646 KB) [file 12885_2014_5109_MOESM1_ESM.tiff]
